# Intradermal delivery of receptor‐binding domain of SARS‐CoV‐2 spike protein with dissolvable microneedles to induce humoral and cellular responses in mice

**DOI:** 10.1002/btm2.10202

**Published:** 2020-12-12

**Authors:** Chaiyaporn Kuwentrai, Jinming Yu, Li Rong, Bao‐Zhong Zhang, Ye‐Fan Hu, Hua‐Rui Gong, Ying Dou, Jian Deng, Jian‐Dong Huang, Chenjie Xu

**Affiliations:** ^1^ School of Biomedical Sciences, Li Ka Shing Faculty of Medicine University of Hong Kong Hong Kong SAR China; ^2^ Department of Biomedical Engineering City University of Hong Kong Hong Kong SAR China; ^3^ Institute of Synthetic Biology, Shenzhen Institutes of Advanced Technology Chinese Academy of Sciences Shenzhen China; ^4^ Department of Medicine University of Hong Kong, Queen Mary Hospital Hong Kong China

**Keywords:** COVID‐19, intradermal delivery, microneedle, receptor‐binding domain, SARS‐CoV‐2, spike protein

## Abstract

The S1 subunit of severe acute respiratory syndrome coronavirus 2 (SARS‐CoV‐2) spike protein contains an immunogenic receptor‐binding domain (RBD), which is a promising candidate for the development of a potential vaccine. This study demonstrated that intradermal delivery of an S‐RBD vaccine using a dissolvable microneedle skin patch can induce both significant B‐cell and significant T‐cell responses against S‐RBD. Importantly, the outcomes were comparable to that of conventional bolus injection.

## 1 INTRODUCTION

The coronavirus disease 2019 (COVID‐19) pandemic due to the severe acute respiratory syndrome coronavirus 2 (SARS‐CoV‐2) has had an unprecedented impact worldwide.^1^, ^2^, ^3^ Effective vaccines are urgently needed to prevent infection/reinfection in the long term and allow life to return to normal. The SARS‐CoV‐2 spike glycoprotein (S protein) plays important roles in viral adhesion, fusion, and entry into cells, and has been identified as a key target for vaccine development. Currently, multiple vaccines based on the SARS‐CoV‐2 spike protein are under evaluation. One candidate is the receptor‐binding domain (RBD) in the S1 subunit of the S protein that specifically binds to angiotensin‐converting enzyme 2 (ACE2) receptor on target cells.^4^ For example, Ravichandran et al recently immunized rabbits with different targets of the SARS‐CoV‐2 S protein and found that immunization with RBD induced high‐affinity antibodies.^5^ There is also an emerging global phase 3 clinical trial testing the efficacy, safety and immunogenicity of **Ad5‐nCoV** (adenovirus type 5 vector expressing S‐RBD).^6^

After a viable vaccine is developed and manufactured, the storage, transportion, and administration of the vaccine will be of prime importance for successfully controlling the pandemic. The efficacy of a vaccine can be influenced by dosage, regimen, site of vaccination, and method of delivery. Most SARS‐CoV‐2 vaccine candidates currently in clinical trials are delivered through intramuscular (IM) injection, including the nonreplicating viral vector vaccine (ChAdOx1‐S) from the University of Oxford/AstraZeneca and the mRNA‐1273 vaccine from Moderna whereas the DNA vaccine from Inovio Pharmaceuticals is delivered through intradermal injection.^7^ Both IM and intradermal injections rely on conventional techniques, but they can have serious limitations including the need for trained professionals to accurately inject the formulation safely and the potential for blood‐related infections.^8^, ^9^, ^10^, ^11^ Therefore, other nonconventional techniques should be considered to minimize repeat doses and the need for trained personnel.

Microneedle (MN)‐based intradermal delivery is an emerging delivery method for vaccines. The skin is an immunologically active tissue and contains antigen‐presenting dendritic cells, Langerhans cells and other cells that can transfer antigens *via* lymphatic drainage to initiate antigen‐specific adaptive immune responses.^12^ Compared with other delivery strategies, MN delivery of vaccines offers advantages such as smaller doses, reduced biohazard waste, and pain‐free and fast vaccination. The MNs can be pre‐formulated and stably stored for extended periods of time at room temperature (RT), which facilitates vaccine usage in developing countries with limited cold chain. This addresses the common problems of vaccine storage stability and distribution challenges. For example, Kim et al recently used dissolvable MNs with carboxymethyl cellulose to deliver SARS‐CoV‐2 S1 subunit vaccines in mice, which induced significantly increased antigen‐specific antibodies by 2 weeks.^13^

Inspired by these pioneering works, this study investigated the potential use of dissolvable MN‐based intradermal delivery of S‐RBD proteins as a potential vaccine for COVID‐19. The MN device was made from a mixture of S‐RBD proteins and low‐molecular weight hyaluronic acid (HA) using the micro‐molding method.^14^, ^15^, ^16^, ^17^, ^18^ HA is a naturally occurring substance in skin with no known side effect and the low molecular weight HA (<50 kDa) quickly dissolves in skin as well. The MN device was effective in penetrating the mouse skin and the resulting immunization elicited significant B cell antibody responses and interferon‐gamma (IFN‐γ)‐based T‐cell responses compared to nonimmunized controls. In contrast to conventional subcutaneous injection, MN‐based intradermal delivery of S‐RBD vaccine is a minimum invasive method that could facilitate rapid control of the COVID‐19 pandemic. However, we discovered that this platform is unsuitable for the delivery of mRNA. For example, we showed that luciferase mRNA embedded in the dissolvable MNs did not induce protein expression comparable to that of bolus injection.

## 2 MATERIALS AND METHODS

### 2.1 Synthesis of S‐RBD protein

The S‐RBD protein domain of the spike protein (amino acid residues 306 to 543) was cloned and purified from *Escherichia coli* as reported. This was formulated at a ratio of 9:1 in aluminum hydroxide gel (InvivoGen).

### 2.2 Transfection reagent preparation

InstantFECT liposomes donated by PGR‐Solutions (Pittsburgh, Pennsylvania)^19^ were prepared by adding the recommended amounts (100–1000 μl) of the reconstitution solution to a dried film. The film was allowed to set for 1 min and then vortexed for 1 min to rehydrate the lipid film into a slightly translucent suspension.

### 2.3 mRNA synthesis

Tobacco mosaic virus (TMV) 5′ and 3′ un‐translated regions (UTR) were cloned into a pUC57 plasmid vector. A copy of a poly‐adenine (A) tail (130 bases in length) was then inserted behind the 3′ UTR to yield the DNA backbone for the mRNA, namely the pRV (Puc‐57 recombinant vector) plasmid. The luciferase reporter gene was inserted into the pRV backbone by gene cloning to obtain pRV‐luciferase plasmids. in vitro transcription of luciferase mRNA from the corresponding plasmid DNAs was performed using a T7‐HiScribe mRNA synthesis kit (NEB). The luciferase mRNAs were synthesized by in vitro transcription (IVT) with a portion of uridine bases (UTP) substituted with N‐methyl pseudouridine triphosphate (TriLink BioTechnologies). The IVT reaction mixture containing plasmid DNA (1–2 μg), nucleoside triphosphates [NTPs] (7.5 mM adenosine triphosphate [ATP], 7.5 mM cytidine triphosphate [CTP], 7.5 mM guanosine triphosphate [GTP], 5 mM uridine triphosphate [UTP], and 2.5 mM N‐methyl pseudouridine), T7 polymerase mix (2 μl), and T7 buffer mix (2 μl) was kept at 37°C for 2 h. The mRNA product was purified by lithium chloride precipitation followed by washing in 70% ethanol. The modified IVT mRNA was then 5′‐capped using the vaccinia‐virus‐capping system (NEB). The 5′‐cap‐modified IVT mRNA products were stored at −20°C.

### 2.4 MN fabrication

MN patches were made using a micro‐molding method. Briefly, HA (molecular weight: 48 K, 100 mg/ml) was dissolved in deionized water (100 mg/ml). Alexa Fluor‐546 rabbit IgG (Z25304, Thermo Fisher) or RBD protein (25 μg) formulated with aluminum hydroxide gel or 5 μg luciferase mRNA formulated with 4 μl InstantFECT liposomes was mixed with the HA solution. Next, 50 μl of the mixture was added to a PDMS negative mold and centrifuged at 4000 rpm for 3 min to ensure all cavities in the mold were filled. After drying overnight at RT, additional HA solution was added to form the backing of the patch. After drying, the patch was peeled off from the mold and preserved in a dry box until use.

### 2.5 Animal experiments

All BALB/c mice were obtained from the Laboratory Animal Unit of the University of Hong Kong. All animal experiments were approved by the Committee on the Use of Live Animals in Teaching & Research, the University of Hong Kong (CULATR 5312‐20). Vaccination was performed using intradermal delivery of MN‐formulated S‐RBD (25 μg/mice) or subcutaneous injection of S‐RBD (25 μg/mice) on Days 0, 3, and 7. Blood samples were drawn from the tail vein on Days 14, 21, 28, 67, and 97.

### 2.6 In vivo imaging

For luciferase mRNA delivery, 24 and 48 h after injection, BALB/C mice were anesthetized with ketamine and dopamine (25:1 ratio). Next, 100 μl of luciferin substrate (30 mg/ml, Gold Biotechnology) was injected intraperitoneally into the mice. After 5 min, all mice were viewed under an in vivo imager (IVIS SPECTRUM) to monitor luciferase signals.

### 2.7 Histological analyses

Paraffin‐embedded skin sections were stained with hematoxylin and eosin (HE) and viewed under a BX51 Olympus Light microscope.

### 2.8 IFN‐γ ELISPOT assay

On Day 28, mice were sacrificed and spleen cells were collected for IFN‐γ ELISPOT analysis. Briefly, 100 μl of spleen cells were incubated on the IFN‐γ ELISPOT plate, which was preactivated for 30 min using 200 μl DMEM media without FBS. Subsequently, 5 μg of the peptide (S‐RBD and the positive inducer) were added and cells were incubated for 20 h at 37°C in 5% CO_2_. After the media was discarded, cells were washed in a washing buffer and incubated with the secondary biotinylated antibody for 1 h at RT. Finally, cells were incubated with the biotin substrate, followed by washing and drying to yield visible spots in positive wells.

### 2.9 Enzyme‐linked immunosorbent assay (ELISA)

At Days 14, 21, 28, 67, and 97 from the first vaccination, blood collected from tail vein was centrifuged at 3000 rpm for 30 min. The separated serum was stored at −80°C until use. A 96‐well ELISA plate was coated with 10 μg/ml of antigen (S‐RBD) in coating medium. Specifically, 100 μl of the solution was added to each well at 4°C overnight. After 12 h, the solution was discarded and the plate was blocked with blocking buffer (5% milk in tris‐buffered saline mixed with tween 20 [TBST]) for 3 h at RT. The wells were washed with TBST six times to remove milk precipitates. Next, serum collected from above was serially diluted in milk‐TBST solution at a ratio of 1:3, 1:12, 1:48, 1:192, 1:768, 1:3072, 1:12288, and 1:49152. The diluted serum was added to the wells and incubated for 1 h at RT. The plate was washed five times in 1X TBST and then incubated with mouse IgG secondary antibody diluted in milk‐TBST solution in a ratio of 1:3000. After incubation for 1 h at RT, the plate was washed six times with 1X TBST. Next, 100 μl of 3,3′,5,5′‐tetramethylbenzidine (TMB substrate) was added to each well and incubated at 37°C for 30 min. Finally, the reaction was stopped with H_2_SO_4_ (50 μl per well) and the absorbance was read at 450 nm. ELISA data were collected on a Varioskan Flash spectral scanning multimode reader (Thermo Scientific).

### 2.10 Statistical analysis

All results were plotted in Prism 7 (GraphPad Software Inc, San Diego, California). Statistical comparisons between groups for ELISA were determined by unpaired nonparametric *t*‐test (Mann–Whitney test) using Prism 7. Statistical comparisons between groups for ELISPOT were determined by unpaired parametric *t*‐test using Prism 7. For all tests, *p* < 0.05 was considered statistically significant.

## 3 RESULTS

### 3.1 Optimization of the protocol for MN patch fabrication

HA was chosen as the building block for the MN device because it has excellent biocompatibility and biodegradability, and is regularly used in cosmetics as a dermal filler.^4^ Several types of HA are available with different molecular weights, and the concentration of HA determines the viscosity of the solution and thus the fabrication process. To optimize these parameters, we chose Alexa Fluor‐546 tagged rabbit IgG as the model protein to prepare the model MNs.

**FIGURE 1:**
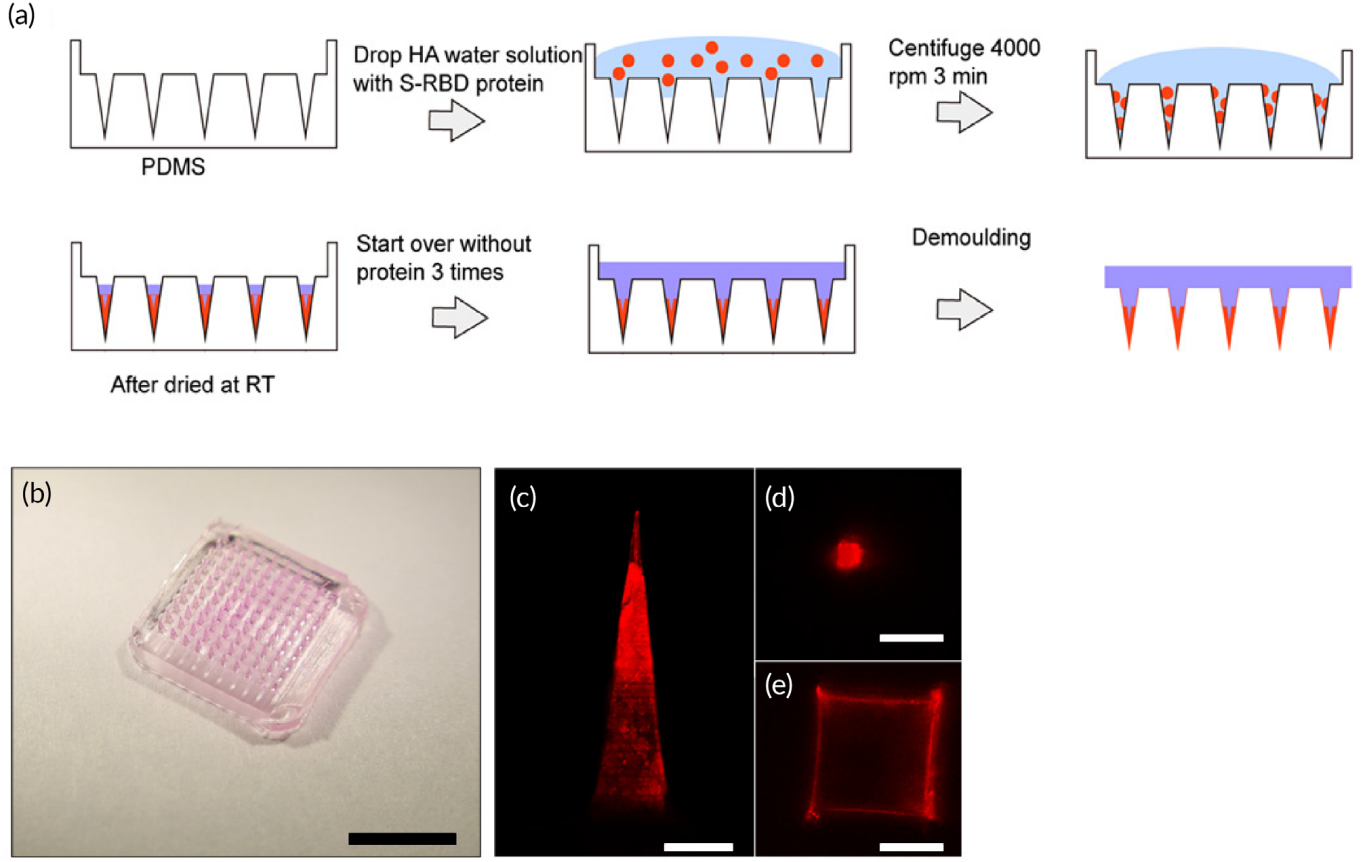
Fabrication and characterization of hyaluronic acid (HA) microneedles (MNs): (a) Illustration of MN fabrication. (b) Images of HA MNs containing Alexa Fluor‐546 rabbit IgG protein (scale bar, 5 mm) (c–e) Confocal images of HA MN tips with Alexa Fluor‐546 rabbit IgG protein at different positions (scale bar c, 200 μm, scale bar d–f, 100 μm)

The MN patch was made through micro‐molding.^20^, ^21^ Firstly, we prepared HA solution with distilled water. There are three types of medical grade HA (i.e., 48, 300, and 1100 K). However, HA with molecular weight larger than 300 K cannot dissolve well in water and forms a super viscous solution even at a concentration of 20 mg/ml. We examined the concentration effect in 48 k HA by preparing 100 and 50 mg/ml solutions, which showed that 100 mg/ml was the highest concentration for a manipulable solution. We used a two‐step procedure: (1) the Alexa Fluor‐546 tagged rabbit IgG protein was used to fill the MN tips and (2) blank HA solution was used to form the backing (Figure 1(a)). The prepared MN patch (Figure 1(b)) can be seen as a fluorescent tip (Figure 1(c–e)) with nonfluorescent backing.

### 3.2 Intradermal delivery of S‐RBD protein using MNs for immunization

**FIGURE 2:**
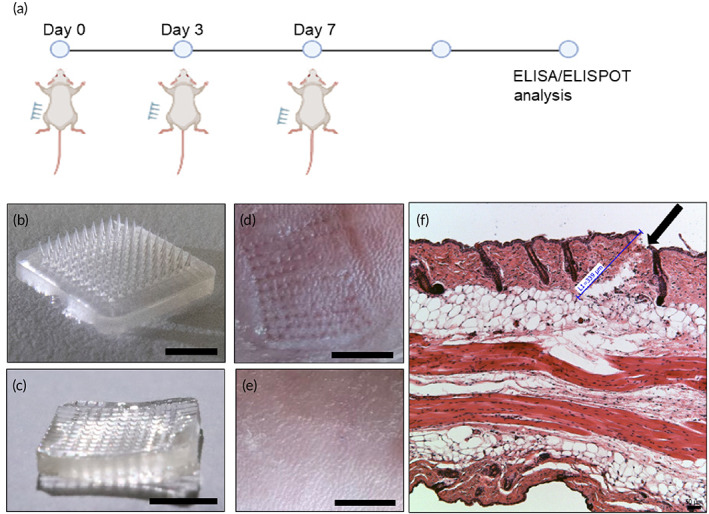
Vaccination with S‐receptor‐binding domain (RBD) microneedles (MNs): (a) Illustration of vaccination process. Image of MN‐RBD patch (b) before injection (tips present) (scale bar, 5 mm) and (c) after injection (tips absent) (scale bar, 5 mm). Image of penetrated mouse skin (d) immediately after injection (scale bar, 5 mm) and (e) 24 h after MN injection (scale bar, 5 mm). (f) Histological image of paraffin‐embedded sections of MN‐penetrated mouse skin. Black arrow indicates site of skin penetration by MNs (scale bar, 50 μm)

Using the optimized protocol above, we made the S‐RBD MN patches containing 25 μg S‐RBD protein per patch (1 × 1 cm^2^ with 100 MN tips). The dosage of protein was determined in our previous study.^22^ Control patches were also made without S‐RBD protein. These HA patches dissolved instantly when they were placed in solution. Both S‐RBD and control patches were administered by a thumb press onto the shaved skin of BALB/c mice at Days 0, 3, and 7 (*n* = 5 per group) (Figure 2(a)). Sufficient force is confirmed when the back part of the MN patch has fully attached to the skin after pressing. As shown in Figure 2(b,c), the MN tips remained in the mouse skin after topical application (10 s). Figure 2(d) clearly shows MN marks on the skin after application, which disappeared after 24 h (Figure 2(e)). Wax‐embedded sections of MN‐penetrated mouse skin (10‐μm thickness) confirmed the MNs penetrated through the epidermis into the dermis layer (Figure 2(f)), as seen by 340‐μm deep holes.

### 3.3 MN‐based S‐RBD immunizations lead to specific B‐cell antibody responses

**FIGURE 3:**
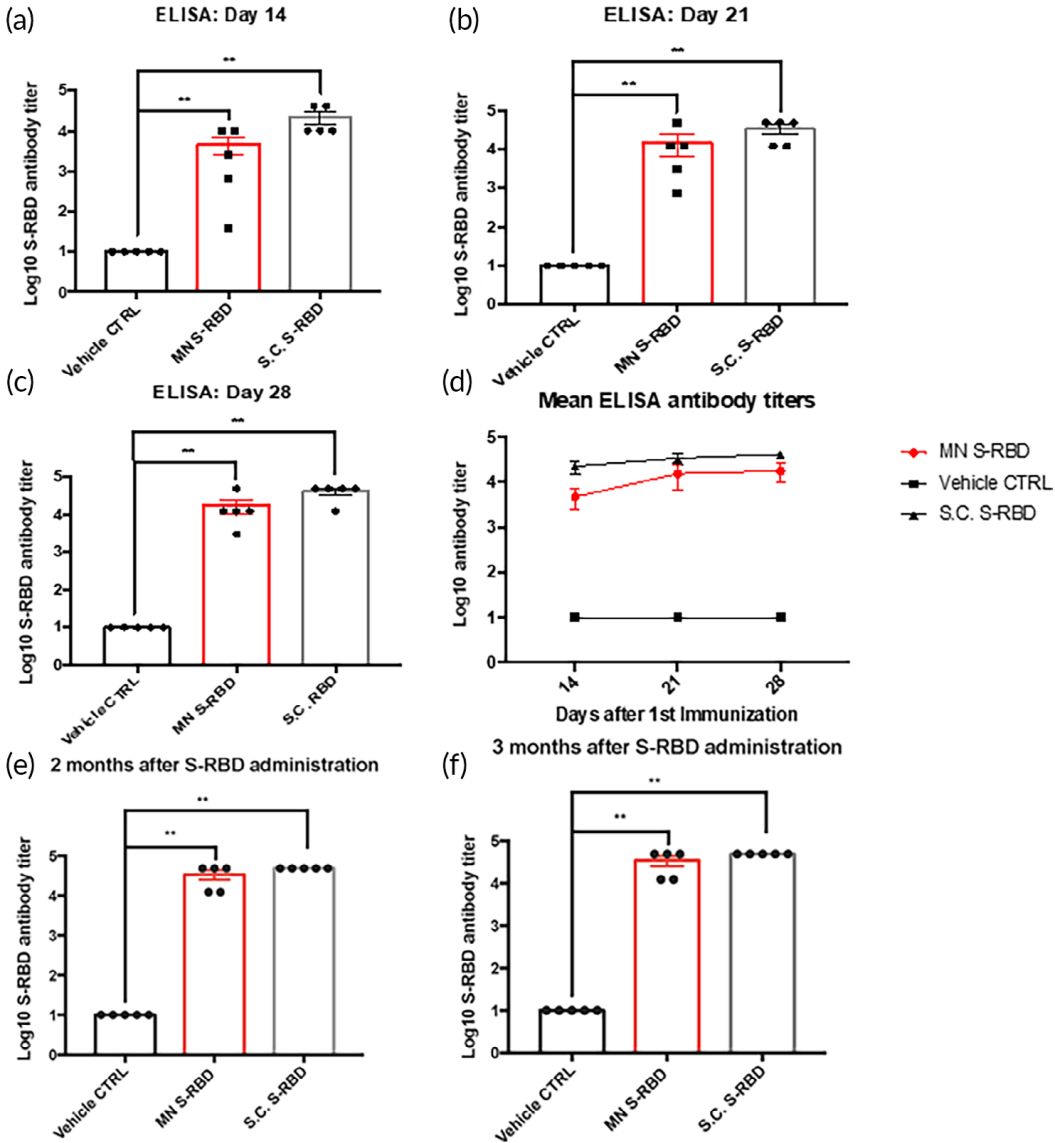
Specific B‐cell antibody responses to vaccination. ELISA results of serum at (a) Day 14, (b) Day 21, and (c) Day 28 post vaccination, showing Log10 antibody titers against S‐receptor‐binding domain (RBD) protein in the S.C. S‐RBD immunization group, S‐RBD microneedle (MN) immunization group, and vehicle control group. ELISA absorbance measurements at 450 nm were normalized to standard cutoff values. Student's unpaired nonparametric *t*‐test (Mann–Whitney) was used with multiple *t*‐test adjustment. Data were expressed as mean ± SEM. *p < 0.05, **p < 0.01, ***p < 0.001, ****p < 0.0001, ns refers to “not significant.” (d) Summary of Log10 Mean S‐RBD antibody titers from S.C. S‐RBD, S‐RBD MN, and vehicle‐control MN groups. (e) ELISA results showing S‐RBD antibody titers in the sera of different mice groups (S.C. S‐RBD group, S‐RBD MN group, and vehicle‐control MN group) at 2 months (Day 67). (f) ELISA results showing S‐RBD antibody titers in the sera of different mice groups (S.C. S‐RBD group, S‐RBD MN group, and vehicle‐control MN group) at 3 months (Day 97)

We investigated the B‐cell antibody response after MN‐based intradermal delivery of S‐RBD protein. The S‐RBD protein was used as the diagnostic antigen in the ELISA assay to detect IgG antibodies against SARS‐CoV‐2 in sera of mice treated with subcutaneously injected (S.C.) S‐RBD (25 μg/mice), MN delivery of S‐RBD (25 μg/mice), or MN delivery of vehicle controls. We performed serum ELISA after three rounds of administration of S‐RBD in BALB/c mice at Days 0, 4, and 7. Sera were collected on Days 14, 21, and 28 from all three groups (S.C. S‐RBD group, S‐RBD MN group, and vehicle‐control MN group). Significant differences were observed in the levels of S‐RBD antibody titers and A450/A630 detected by ELISA detection between the S‐RBD MN group or S.C. S‐RBD group compared to the vehicle‐control MN group. In the S‐RBD MN group, the difference in S‐RBD antibody titers could be seen at Day 14 after the first immunization, which increased at Day 21, and further increased to a maximum antibody titer of 4.9 × 10^4^ at Day 28 (Figure 3(a–c)). By Day 28, an average antibody titer of over 1.7 × 10^4^ was detected in the S‐RBD MN groups, indicating the MN delivery of S‐RBD was highly efficient, and the immunization generated specific S‐RBD antibodies in mice blood. The S.C. S‐RBD group exhibited comparable S‐RBD antibody titers to that of the S‐RBD MN group (Figure 3(d)). These findings showed there was a strong antibody response against RBD, indicating MN‐based S‐RBD immunizations can induce B‐cell immunity against SARS‐CoV‐2. Furthermore, we assessed the longevity of the antibody responses in mice immunized from the 3 groups (S.C. S‐RBD group, S‐RBD MN group, and vehicle‐control MN group) at 2 months and 3 months after the final vaccination (Figure 3(e,f)). Both the S.C. S‐RBD group and the S‐RBD MN groups exhibited same or higher S‐RBD specific antibody titers by 2 and 3 months compared to the vehicle‐control MN group.

### 3.4 MN‐based S‐RBD immunizations lead to specific T‐cell responses

**FIGURE 4:**
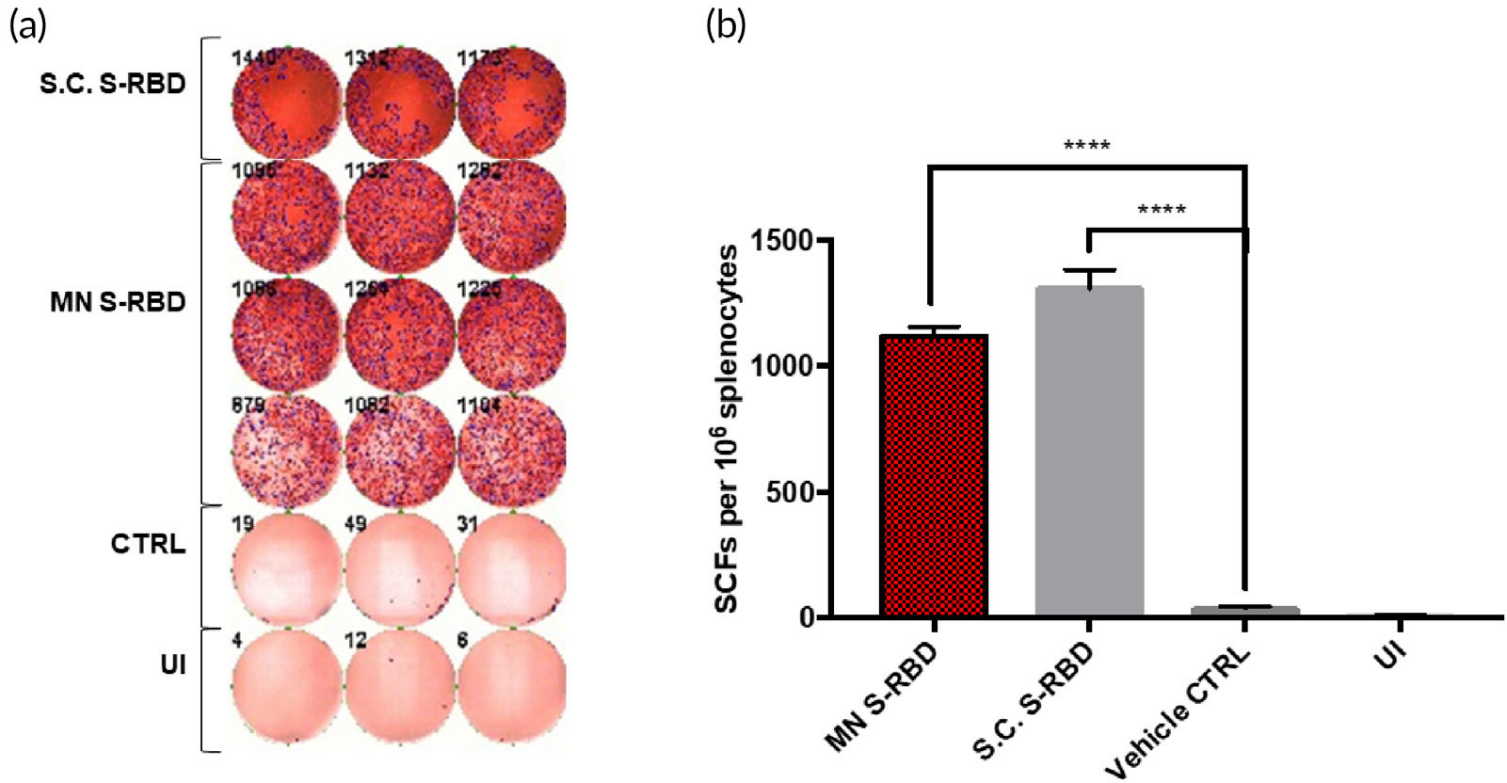
Interferon‐gamma (IFN‐γ) T‐cell responses: (a) IFN‐γ ELISPOT plate counts for S.C. S‐receptor‐binding domain (RBD) stimulated, microneedle (MN) S‐RBD stimulated, blank MN stimulated (CTRL) and un‐induced (UI) groups. (b) Bar chart shows mean IFN‐γ ELISPOT counts for all four groups. Student's unpaired parametric *t*‐test was used with Welch's correction. Data were expressed as mean ± SD. *p < 0.05, **p < 0.01, ***p < 0.001, ****p < 0.0001, ns refers to “not significant”

We investigated the T‐cell IFN‐γ response in mouse splenocytes to immunization by S.C. S‐RBD, S‐RBD MN, and vehicle‐control MN by ELISPOT using S‐RBD protein (5 μg/ml) as the stimulator. Ionomycin was used as the positive inducer and FBS‐free medium was used as the negative inducer to check whether MN‐based S‐RBD immunizations could provoke T‐cell activation. Figure 4(a,b) shows significant IFN‐γ was released by T cells by Day 29 in the S.C. S‐RBD and S‐RBD MN groups compared to vehicle and noninduced controls. The high levels of IFN‐γ producing T cells may produce strong antiviral protection against SARS‐CoV‐2. We also noticed that the S.C. S‐RBD group had levels of IFN‐γ producing T cells comparable to the S‐RBD MN group (Figure 4(b)).

### 3.5 MN delivery of mRNA

**FIGURE 5:**
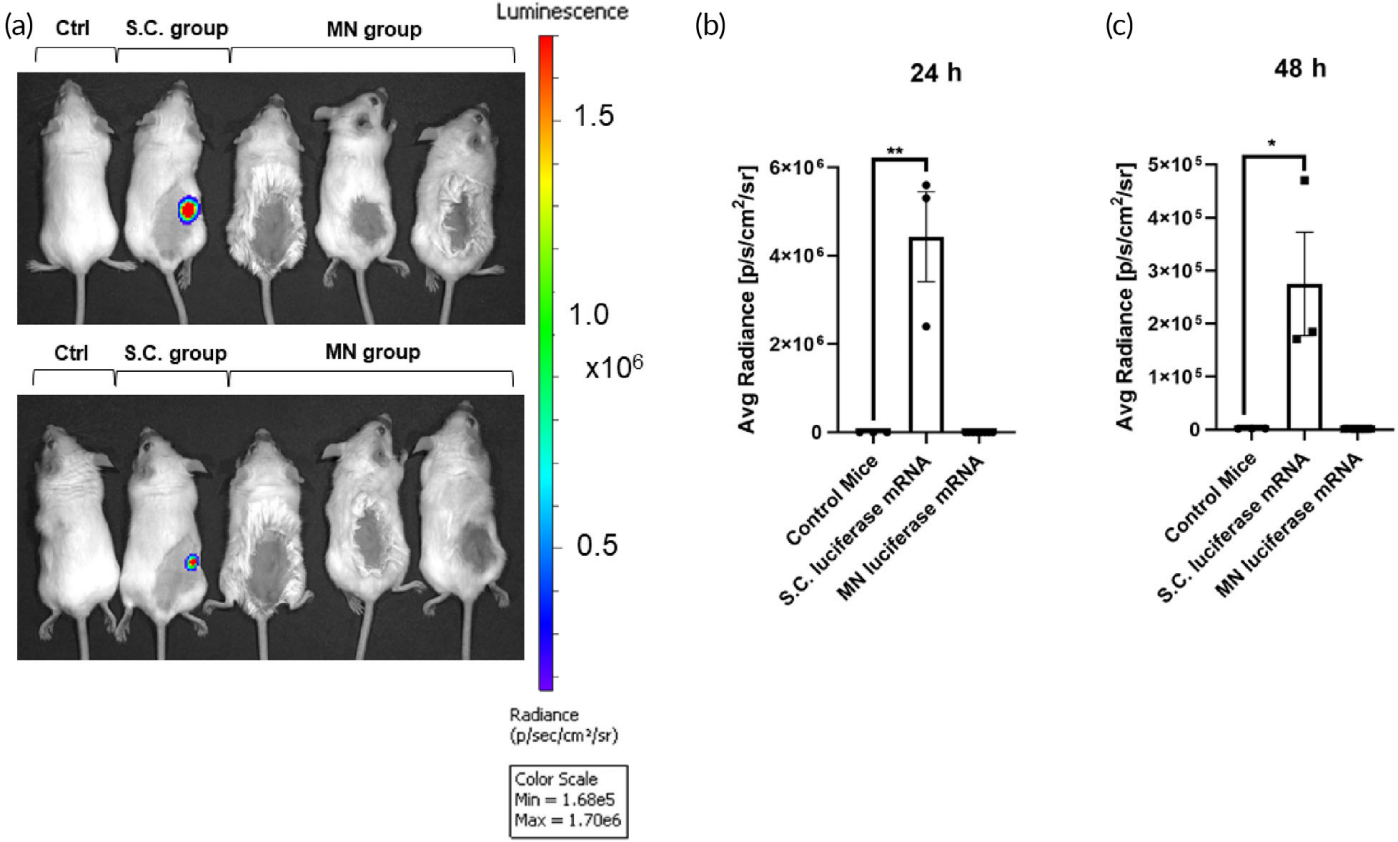
Hyaluronic acid (HA) microneedles (MNs) were unable to deliver mRNA: (a) in vivo imaging of BALB/C mice 24 and 48 h after luciferase mRNA (S.C.), and MN‐based luciferase mRNA administration compared to control. Comparison of average radiance [p/s/cm^2^/sr] between luciferase mRNA (S.C.) group, MN‐based luciferase mRNA group and control group (b) 24 h and (c) 48 h after initial mRNA administration. Significance of mean average radiance [p/s/cm^2^/sr] between groups was determined by unpaired parametric *t*‐test, *p* < 0.05

Besides protein‐based vaccines for COVID‐19, another potential type of vaccine are mRNA vaccines like mRNA‐1273 from Moderna. To test the MN system for the delivery of mRNA, we used the above protocol to produce HA MNs containing luciferase mRNA. BALB/C mice were either subcutaneously injected with 5 μg of luciferase mRNA with liposomes or topically administered luciferase mRNA MNs. For subcutaneous injection, luciferase mRNA was stored in −20° until 30 min prior to injection where it is mixed with liposome before injection. After 24 and 48 h, mice were anesthetized and given a luciferin substrate (I.P.) before imaging (Figure 5(a)). Subcutaneously injected luciferase mRNA successfully expressed luciferase enzymes after 24 h and the luciferase activities were significantly decreased at 48 h (Figure 5(b,c)). Unfortunately, MN delivery of luciferase mRNA did not produce significant luciferase compared with the average radiance scores of nonchallenged control mice at 24 and 48 h. However, we cannot rule out degradation of mRNA during the mold fabrication process at RT, which will require further investigation.

## 4 DISCUSSION AND CONCLUSION

This study explored the potential intradermal delivery of S‐RBD proteins using dissolvable HA MNs. The S‐RBD proteins were embedded in the HA MN tips together with an aluminum hydroxide gel adjuvant. The immune response in mice after MN‐based delivery of S‐RBD was analyzed by ELISA and ELISPOT. Specific B‐cell antibodies and IFN‐γ T‐cell responses were detected, suggesting MN‐based S‐RBD could be a potential vaccine formulation for COVID‐19.

There is emerging evidence for the use of MN‐based delivery of vaccines for COVID‐19.^23^ Kim et al demonstrated the delivery of recombinant coronavirus vaccines (SARS‐CoV‐2‐S1 and SARS‐CoV‐2‐S1fRS09 subunit) using carboxymethyl cellulose‐based MN delivery platforms, which were able to induce high titers of SARS‐CoV‐2‐S1 specific antibodies as detected by ELISA. Our team explored the use of an HA‐based MN delivery platform to deliver S‐RBD (SARS‐CoV‐2‐S1) in mice. We obtained similar significant antibody responses, for a longer time up to 97 days after administration. Different from their study, we performed additional ELISPOT analysis that showed significant IFN‐γ T‐cell responses. Specific T‐cell IFN‐γ responses toward viral antigens have been reported as key antiviral protective factors that may participate in the eradication of COVID‐19.^22^ Moreover, T‐cell responses may be equally as important as the induction of specific B‐cell antibodies against SARS‐CoV‐2.^24^, ^25^ Nonetheless, our results for HA‐based MN delivery of luciferase mRNA only showed very weak luminescence signals compared to subcutaneous injection of luciferase (Figure 5). This is a current limitation of this MN delivery system.

The MN‐based intradermal delivery is a unique method for the delivery of vaccines in a noninvasive manner. The whole device is made of biocompatible and biodegradable materials so there would not be any damage to the environment after the disposal, which can be done in these resource‐poor settings. The deployment of tips from this device took 10 s, which facilitates the fast vaccination.

However, there was high variation in the titers of specific S‐RBD antibodies produced by the MN method compared to the S.C. injection method (Figure 3). Importantly, IFN‐γ T‐cell responses were comparable between the S‐RBD MN group and the S.C. S‐RBD group (Figure 4), and both treatment groups showed significant differences from the vehicle‐control MN group. Several factors may limit the efficiency of the MN delivery system for S‐RBD, such as possible loss of antigen activity during the MN formulation. The HA MN platform was unable to deliver mRNA, as shown through the delivery of luciferase mRNA, which did not produce significant expression of the target protein in vivo.

Finally, considering the clinical translation of this technology, there are a few other key issues to solve in the next stage. Regulatory bodies tend to check for sterility for any medical device like this MN vaccine. Considering the antigen and HA properties, this device cannot be sterilized using steam, ionizing radiation (gamma and electron‐beam radiation), and gas (ethylene oxide, formaldehyde) post the fabrication. One possible solution is to fabricate the MN vaccines in a germ‐free production laboratory site. Of course, this idea has to be supported by the regulatory bodies. In addition, it is also important to achieve a cost‐effective fabrication of this device in the large scale. Method in this article involves the use of PDMS molding, centrifugation, drying, and peeling process. This needs well‐trained professionals and multiple equipment, which are expensive. This issue might be solved with 3D printing in the future. Finally, S‐RBD MN in this study was applied into the skin through thumb press, which is not ideal for clinical practice. An applicator that ensures consistent and accurate deployment of MNs is needed.

We hope to address the above limitations in our future study to optimize the HA MN delivery as a non‐invasive system for vaccines, particularly for COVID‐19.

### PEER REVIEW

The peer review history for this article is available at https://publons.com/publon/10.1002/btm2.10202.

## Supporting information


**Appendix S1** Supporting Information.

## Data Availability

The data that support the findings of this study are available from the corresponding author upon reasonable request.
