# Supplementary material for: Intradermal delivery of receptor‐binding domain of SARS‐CoV‐2 spike protein with dissolvable microneedles to induce humoral and cellular responses in mice
Source: Bioeng Transl Med. 2020 Dec 12;6(1):e10202. doi: 10.1002/btm2.10202 (PMC7744900; doi:10.1002/btm2.10202)
Supplement: Supplementary file 1 — Appendix S1 Supporting Information. [file BTM2-6-e10202-s001.docx]

Supplementary materials

Chaiyaporn Kuwentrai^1, #^, Jinming Yu^2, #^, Li Rong^1^, Bao-zhong Zhang^1, 3^, Ye-fan Hu^1, 4^, Hua-rui Gong^1^, Ying Dou^1^, Jian Deng^1^, Jian-Dong Huang^1, 3*^, Chenjie Xu^2*^

^1^ School of Biomedical Sciences, Li Ka Shing Faculty of Medicine, University of Hong Kong, 21 Sassoon Road, Hong Kong SAR, China

^2^ Department of Biomedical Engineering, City University of Hong Kong, 83 Tat Chee Avenue, Kowloon, Hong Kong SAR, China

^3^ Institute of Synthetic Biology, Shenzhen Institutes of Advanced Technology, Chinese Academy of Sciences, 1068 Xueyuan Avenue, University Town, Nanshan, Shenzhen, 518055, China

^4^ Department of Medicine, University of Hong Kong, 4/F Professional Block, Queen Mary Hospital, 102 Pokfulam Road, Hong Kong, China

^#^ Equal contribution.

^*^Correspondence to [chenjie.xu@cityu.edu.hk](mailto:chenjie.xu@cityu.edu.hk); [jdhuang@hku.hk](mailto:jdhuang@hku.hk)

To investigate the storage stability of our microneedle vaccines, we have stored our S-RBD microneedle vaccines for one month at 25 °C in a dehumidifier chamber after production in our lab. We then performed serum ELISA at day 28 after three rounds of administration of either freshly prepared S-RBD microneedle vaccine, 1 month stored S-RBD microneedle vaccine or vehicle control in BALB/c mice at day 0, 4 and 7 (n=5 per group). Significant differences were observed in the levels of S-RBD antibody titers and A450/A630 detected by ELISA detection between the freshly prepared S-RBD MN immunization group or 1 month stored S-RBD MN immunization group compared to the vehicle-control MN group. These results also show that 1 month storage of MN S-RBD does not affect the activity of S-RBD to induce specific S-RBD antibodies in mice when compared to freshly prepared S-RBD microneedle vaccines (Fig S1).

Fig. S1. Specific B cell antibody responses to vaccination. ELISA results of mice serum at day 28 post vaccination, showing Log10 antibody titers against S-RBD protein in the freshly prepared MN S-RBD immunization group, 1 month stored MN S-RBD immunization group and vehicle control group. ELISA absorbance measurements at 450 nm were normalized to standard cut-off values. Student’s unpaired non-parametric *t*-test (Mann-Whitney) was used with multiple *t*-test adjustment. Data were expressed as mean ± SEM. **P* < 0.05, ***P* < 0.01, ****P* < 0.001, *****P* < 0.0001, ns refers to “not significant”.
